# Do the key functions of an intervention designed from the same specifications vary according to context? Investigating the transferability of a public health intervention in France

**DOI:** 10.1186/s13012-019-0880-8

**Published:** 2019-04-02

**Authors:** Mélanie Villeval, Emilie Gaborit, Florent Berault, Thierry Lang, Michelle Kelly-Irving

**Affiliations:** 1 UMR1027, Université de Toulouse, UPS, Inserm, Toulouse, France; 2 Hôpital Purpan, Centre Hospitalo-Universitaire Toulouse, Toulouse, France; 3 Cresco, Université de Toulouse, UPS, Toulouse, France; 4 IFERISS, Université de Toulouse, UPS, Toulouse, France; 5 EQUITY research team-Inserm Unit of Epidemiology and Public Health, Faculté de Médecine, Université Toulouse III, 37 Allées Jules Guesde, 31000 Toulouse, France

**Keywords:** Public health, Interventions, Transferability, Nutrition, School, Key function, Form, Context

## Abstract

**Background:**

The processes at play in the implementation of one program in different contexts are complex and not yet well understood. In order to facilitate both the analysis and transfer of interventions, a “key functions/implementation/context” (FIC) model was developed to structure the description of public health interventions by distinguishing their potentially transferable dimensions (their “key functions”) from those associated with their translation within a specific context (their “form”). It was used to describe and compare preschool preventative nutrition interventions routinely implemented across three territories, in accordance with same national specifications.

**Methods:**

The interventions were independently described by researchers and intervention’s implementers using the FIC model, during several workshops. Their key functions were then classified into 12 themes and compared to assess the extent to which the three interventions were similar.

**Results:**

Despite being produced from the same set of specifications and having similar objectives, the key functions of the interventions in the three departments mostly reflected the same major themes, they did not overlap and were in some cases very different. In one of the three departments, the intervention was markedly different from those of the other two departments. The historical context of the interventions and the specificities of the local actors appear highly determinant of the key functions described.

**Conclusions:**

For the interventions that we studied, some of the key functions varied greatly and translated different concepts of health education and modes of intervention to the population. It now seems vital to improve the description of interventions on the ground in order to highlight the key functions on which they are based, which still often remain implicit. The FIC model could be used to complement other models and theories focusing on the description of the implementation process, its determinants or its evaluation. Its interest is to provide a structure for joint reflection by various actors on the transferable aspects of an intervention, its form and its interactions with the context, in order ultimately to analyse or to improve its potential transferability.

## Background

The transferability of an intervention to different contexts poses many questions and challenges. Public health interventions are not simply technical procedures, applicable irrespective of context; they are more comparable to events occurring within complex systems [1, 2]. In this conceptualisation, context plays a central role in the production of effects induced by an intervention. However, the context is often represented as an external framework, a simple “receptacle” for interventions, or considered merely as one of many factors explaining why an intervention has failed upon implementation [3]. Unless we take the complexity of context/intervention interactions into account, an intervention that is effective in a certain context is unlikely to be “transferable”, i.e. to produce the same results elsewhere [4].

The processes at play in the implementation of one program in different contexts are complex and not yet well understood. One of the core debates within the field of implementation research centres on the tension between fidelity (i.e. the extent to which an intervention is implemented as intended, or replicates an initial intervention) and adaptation or reinvention of interventions [5–7].

Differences observed between interventions produced from a single program and implemented in various contexts may consist of the inevitable and necessary modifications required to adapt the program to its context. However, they may also consist of genuine transformations of the theories underlying the initial program. The tension between fidelity and adaptation is not only a technical matter but also conceptual and political. It is underpinned by fundamental differences in the conceptualisation of public health interventions: while arguments in favour of fidelity tend to rely on RCT language, arguments in favour of adaptation often rely on organisational change and innovation theory [8].

However, a lot of authors in population health intervention field consider that it is essential to combine both fidelity and adaptation [7, 9–11].

A recent literature review on transferability criteria, facilitators and barriers [11] shows that the possibility of adapting an intervention while keeping the primary intervention’s fundamental nature is a strong transferability criterion. This literature analysis shows that the analysis of essential core elements of an intervention’s effects is essential to adapt the intervention to the context in a relevant way, and to achieve the intervention’s implementation [11]. According to the authors, the intervention’s core elements or principles can be defined by ‘theory’, by ‘experience in implementing the intervention’ or by a ‘formal component analysis’ [11].

Also, Pérez et al. emphasise the importance of making the *functioning principles* of the intervention explicit in order to take both fidelity and adaptation into account when describing and evaluating the implementation of an intervention [7].

The challenge in reconciling fidelity and adaptability lies also in the possibility of better using and promoting the expertise of local professionals and their knowledge of the implementation process of interventions and the context in which they are operating [5, 9]. In practice, many interventions are developed and implemented without being evidence-based or scientifically evaluated [12]. However, the processes at work and the theoretical hypotheses underlying these interventions, as well as their implementation and their unexpected effects, can be evaluated. This evaluation process enables knowledge production, by including all stakeholders [12].

In order to facilitate identification of core components of intervention and possibility of intervention’s adaptation, by including local actors in the reflection, a “key functions/implementation/context” (FIC) model was developed and refined [13–15]. It was inspired by Hawe’s research, who stated that it is possible to standardise an intervention implemented in different contexts using its “key functions”, rather than imposing the same form on each of these contexts. This would enable the adaptation of the intervention based on these various contexts while maintaining the fidelity of the intervention [16]. Based on this notion, “Fidelity resides in the theory of the change process, rather than in any particular technology, component, or delivery channel per se. Thus, the role and meaning behind a particular component, rather than its face value, are what matter.” [16].

**Fig. 1:**
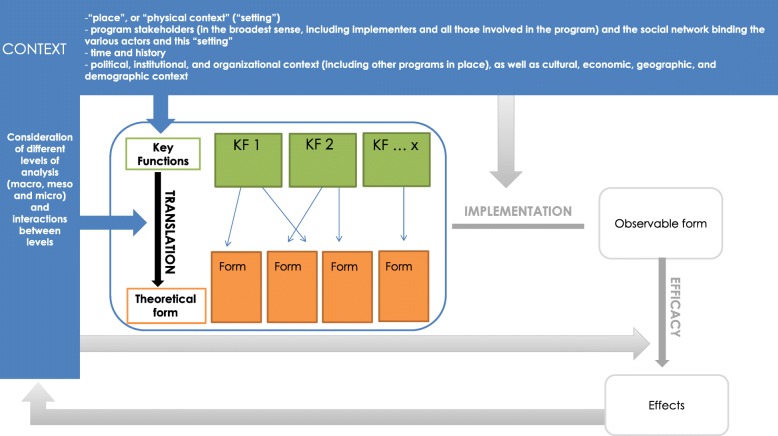
“Key functions/implementation/context” (FIC) model, refined, September 2017

The FIC model can be used to structure the description of public health interventions by distinguishing their potentially transferable dimensions (their “key functions”) from those associated with their translation within a specific context (their “form”). The refined FIC model incorporates a description of the context, which influences both the choice of key functions and the “theoretical” form of the intervention. The observable form that the intervention takes when implemented in practice, and the effects (whether expected or not) that it produces, are observed. In return, the intervention will, itself, change the context (see Fig. 1).

The FIC model is based on the hypothesis that describing an intervention by making these various dimensions explicit enables its key functions to be reproduced during transfer, rather than its form, which can be varied to suit the context in which the functions are to be reproduced. This improves the potential transferability of an intervention, while taking into account context and its role in the production of a public health intervention’s effects.

The purpose of this research was to analyse the extent to which interventions implemented by local agents, or “actors”, using the same national set of specifications would reflect the same intervention theory. Or, conversely, whether differences in form would go beyond local adaptation and involve actual differences in their key functions. The focuses of our analysis were three preschool preventative nutrition interventions routinely implemented across three departments (*départements*) within the same region in France, in accordance with national specifications produced by the National Employee Health Insurance Fund (*Caisse nationale d’assurance maladie des travailleurs salariés*, CNAMTS).

The secondary objective was to confirm the feasibility and usefulness of the FIC approach for describing these interventions in association with local actors.

## Methods

### The Transferiss project

The Transferiss research project (2014–2017) examined the transferability of interventions using a multidisciplinary (political science, sociology and public health) approach. The protocol for this project has been described elsewhere [17]. The description of the interventions is recorded in the “public health” section of this project. The dynamics between actors and the observable implementation of the intervention are described in the “sociology” section of the project. On a completely different scale of context, an analysis of the content of national nutrition-related public health plans and programs and their translation at the regional level was performed as part of the “political sciences” section of the research.

### The preventative nutrition interventions studied and the national specifications

**Fig. 2:**
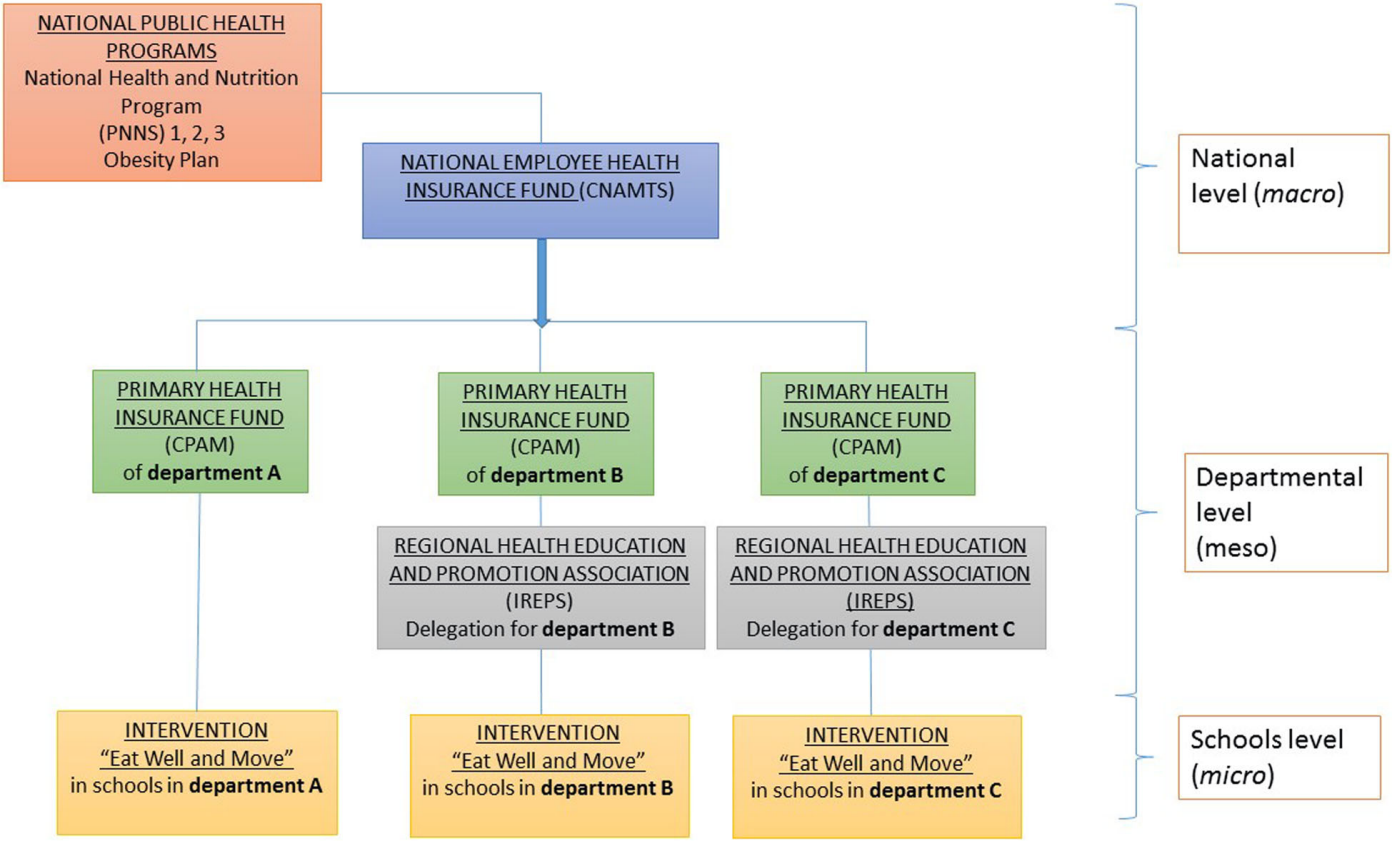
The national nutritional programs, the managing institutions and the “Eat and Move Well!” interventions relationships

The nutrition education interventions studied (“Eat and Move Well!” interventions) aim to prevent overweight and obesity in preschool children. Initiated between 2003 and 2007, they were implemented in three departments of the Occitanie region, anonymized here as departments A, B and C. They form part of several France-wide public health programs (including the National Health and Nutrition Program (PNNS) and the Obesity Plan). They are funded by the CNAMTS via an annual call for projects accompanied by a set of specifications (see Fig. 2). Depending on the department, the interventions are managed by different institutions, but their local implementation is carried out by dieticians, in association with kindergarten teachers.

### The description of the interventions

The key functions, theoretical form and context of the three “Eat and Move Well!” interventions were described using the FIC model (see Fig. 1). Here, the description of each intervention (in departments A, B and C) is centered on their key functions, their translation into a particular form, and their context.

- Based on research by Hawe et al. [1], we define key functions as processes, based on a rationale (more or less explicit), aimed at producing a change in order to address a situation deemed problematic (by public health professionals, researchers or community members, for example). These key functions are applied (by these same stakeholders) according to the context in which this situation has arisen and are translated in a specific form tailored to this context.
- The “form” of the intervention results, therefore, from the process of translating these key functions in a manner appropriate to a particular context. However, this “theoretical” form may be modified during the process of implementing the intervention, producing the “observable” form that the intervention actually takes. This implementation process can be defined as “the process through which interventions are delivered, and what is delivered in practice” [18].
- Context is a vast and complex concept, with ill-defined limits [19]. Given the current lack of a stabilised conceptual framework, describing the context of public health interventions remains an exploratory and imperfect exercise. To avoid reducing it to a simple “backdrop” to the program, while describing it in operational terms, the decision was made to define it based on several dimensions: the physical context, or “setting” in which the intervention is implemented, the set of stakeholders involved and the social network binding these actors, time and history [1], and the institutional, organisational, cultural, economic context etc., at local, regional and national levels. Attention was given to the way in which these different dimensions interact with the intervention.

As such, as the FIC model shows, the effects (expected and unexpected) are not only produced by the theoretical intervention but by all the interactions between context and intervention (choice of key functions, translation into a specific form and implementation of the intervention).

The description process was based on the collation of various types of knowledge—multidisciplinary academic knowledge, professional knowledge and experiential knowledge:

Within this framework, interviews and workshop meetings were organised with the actors implementing the interventions by MV, PhD. Some participants already knew the researcher, since they had worked together in a previous research project. All of them were aware of the project goals and methods. Every implementer of the ‘Eat and Move Well!’ intervention in departments A, B and C was invited to participate in a comprehensive manner, by email and/or telephone. Interviews and workshop meetings were mostly conducted at the participant’s workplaces and lasted between three-quarters of an hour and 2 h and a half. At the first meeting, a semi-structured exploratory individual or group interview was conducted with them, in which various topics were discussed: the activities and objectives of the program, the strategies for achieving these objectives, the evolution of the program over time, the relationships between the stakeholders, the level and manner of adaptation according to schools/teachers, what remains constant despite these adaptations, resources, opportunities and challenges of implementation, and consideration of social inequalities in health. Analysis of these interviews was used to produce a preliminary version of the FIC description of the interventions and a working guide for subsequent meetings with the implementing actors, which allowed to refine the FIC description over time.

In total, two interviews and three working meetings with two dieticians and a sports teacher were conducted in department A, and two interviews and three working meetings with two dieticians were conducted in department B. In department C, on the other hand, only two interviews could be conducted with two dieticians, with no subsequent meetings to refine the description. As such, while the description produced in departments A and B was genuinely co-constructed by the actors on the ground and the researcher, in department C the description was primarily produced by the public health researcher based on interviews conducted with actors in this area. One of the dieticians implementing the intervention retired after our first interview and the other dieticians did not respond to our requests to rework the description of the intervention with them. Interviews and workshop meetings were audio-recorded and field notes were made. Recordings were transcribed to assist data analysis.

In order to refine the description of the contexts in which the interventions are implemented in the three departments and to better understand the links between intervention and context, five interviews were also conducted with seven local institutional actors (intervention managers and promoters), and one interview was conducted with two actors at national level (CNAMTS). These interviews were conducted by MV and EG, using a specific interview guide.

The tools were used by the stakeholders during the planning, coordination and implementation phases of the intervention, as well as the teaching materials used to deliver the *“Eat and Move Well!”* interventions to the pupils. Analysis of these documents allowed an initial comparison of the departments studied in terms of the number of tools, the use made of them, and the status accorded to them (teaching materials used for implementation purposes, and program coordination and monitoring tools). The analysis was used to contribute to discussions with the implementing actors and featured in debates on elements belonging to the form or the key functions of the interventions.

Data analysis was conducted by MV, but regular interactions with EG, in charge of the “sociology” section of the project, provided a critical appraisal and enhanced the description of the interventions. As part of the sociology section, the results of which are published elsewhere [20], 31 interviews and 255 h of observation of the intervention in practice and of coordination meetings were analysed, and a field notebook was kept.

### The comparison between the interventions

Once the interventions had been described using the FIC model, the common themes of the key functions of these three interventions were defined. These themes were chosen because they have a higher level of generality than the key functions, facilitating the comparability of the interventions. All the key functions were then categorised into 12 themes: participation of schools in the program, diagnosis of needs and context, delivery of nutrition education sessions to pupils by dieticians, interaction with teachers, adaptation to school context, promotion of physical activity, comprehensive action, program continuity, interaction with parents, interaction with other actors, screening for and management of overweight and obesity, and coordination with other prevention campaigns. Using this thematic classification system, three levels of similarity were defined between the key functions of the three departments:

- Key function is identical to the key function of one or two other interventions.
- Key function is similar to the key function of one or two other interventions, but with slight differences.
- Key function is unique or very different from the key functions of the other interventions.

## Results

### Description of the three interventions using the FIC model

**Fig. 3:**
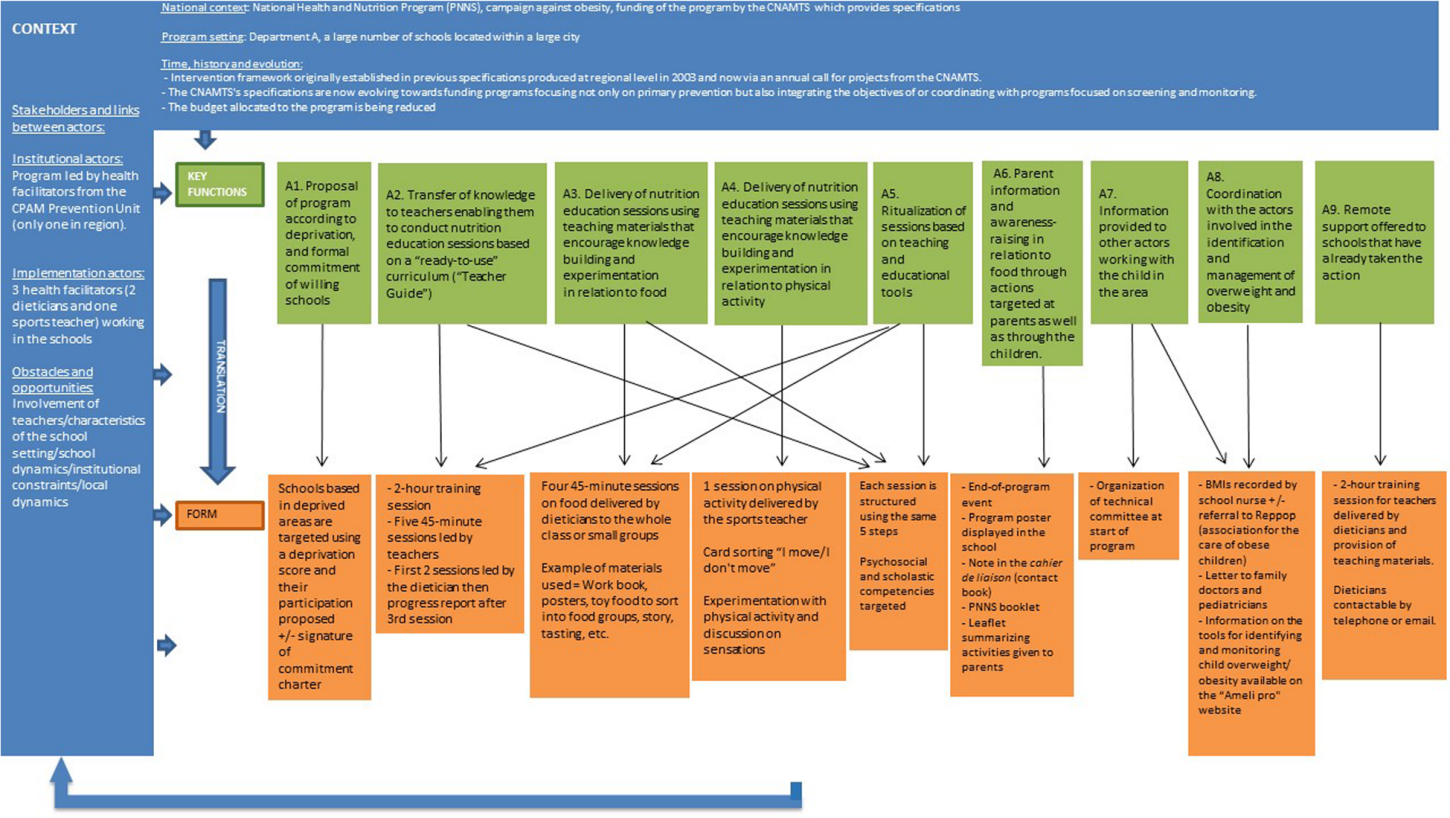
Description of the “Eat Well and Move” intervention in department A using the FIC model

**Fig. 4:**
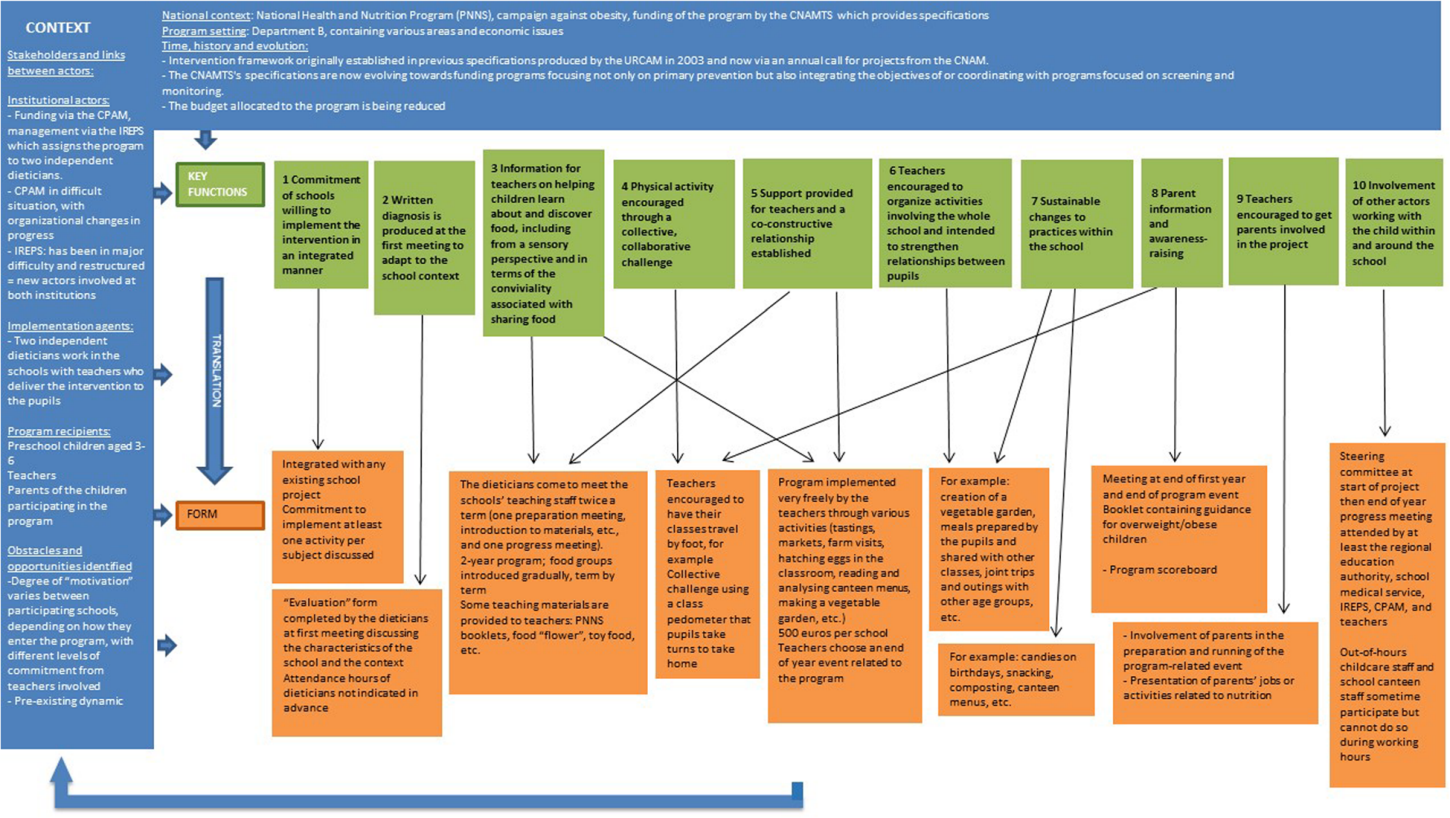
Description of the “Eat Well and Move” intervention in department B using the FIC model

**Fig. 5:**
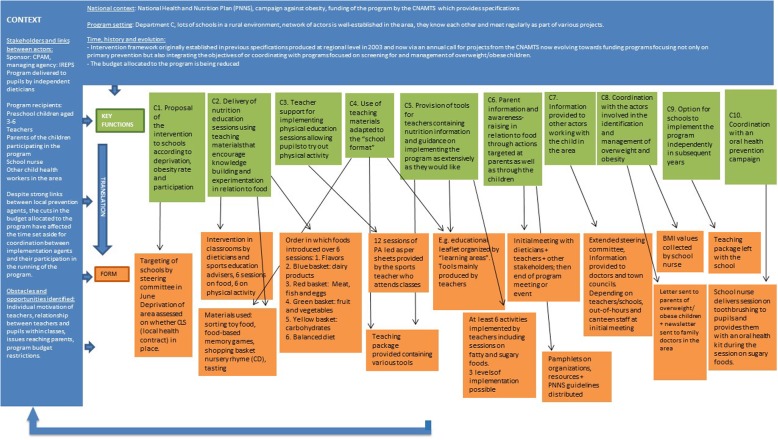
Description of the “Eat Well and Move” intervention in department C using the FIC model

In department A, 9 key functions were described, while 10 key functions were described in departments B and C. These descriptions are summarised as diagrams in Figs. 3, 4 and 5.

### Comparison of the interventions

Regarding the forms, our results show that the forms of the intervention vary across the three departments. We see, for example, that the number and content of the intervention tools are different, as well as the number of nutrition education sessions run and the order in which the food groups are discussed by the dieticians. Many “forms” of the intervention are specific to just one of the departments (e.g. making a vegetable garden, tooth brushing sessions). In particular, we can see that department B, where dieticians only interact with teachers, differs from departments A and C where the dieticians interact directly with the pupils. Furthermore, in this department, the program is conducted over 2 years, whereas in the other two areas it lasts for just one school year. But, do these specificities of form correspond to actual differences of key functions?

Regarding the key functions, the comparison between the key functions of the “Eat and Move Well!” interventions in departments A, B and C is summarised in the following table (Table 1).

**Table 1:**
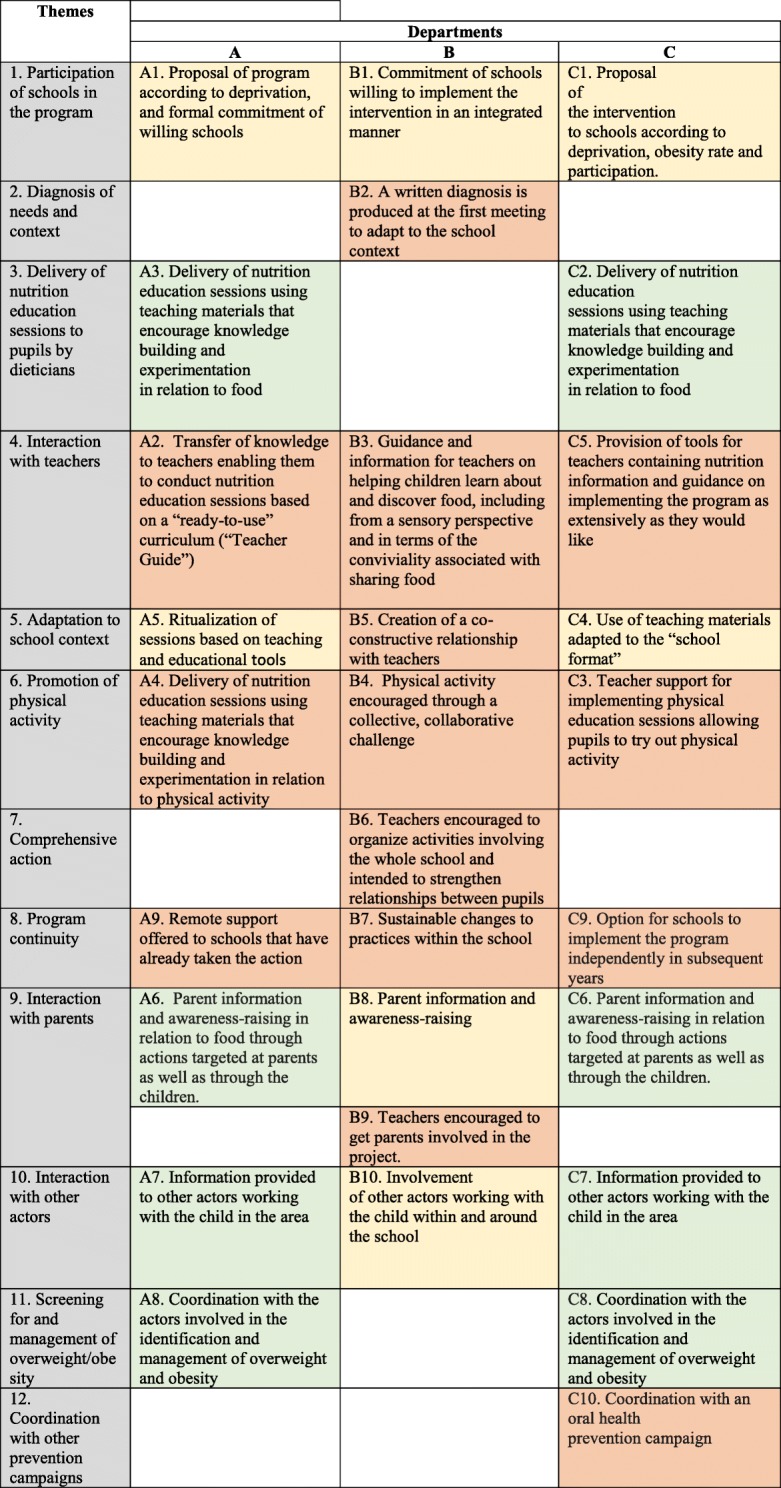
Comparison between the key functions of the “Eat and Move Well” interventions in departments A, B and C, based on themes identified

Key

For the same theme:

Key function is identical to the key function of one or two other interventions

Key function is similar to the key function of one or two other interventions, but with slight differences

Key function is unique or very different from the key functions of the other interventions

Table 1. Comparison between the key functions of the “Eat and Move Well” interventions in departments A, B and C, based on themes identified.

In Table 1, we see that the key functions of the three interventions do not overlap. The interventions in departments A and C contain many similar or identical key functions (*n* = 7 in each of these departments), while the intervention in department B contains mainly “unique” or “very different” key functions from those of the other departments’ interventions (*n* = 7). The themes “Nutrition education sessions delivered to pupils by dieticians” and “Screening for and management of overweight/obesity” constitute identical key functions in departments A and C, but the department B intervention contains no such key functions.

In addition, two themes are specific to the department B intervention (“Diagnosis of needs and context” and “Comprehensive action”), as opposed to just one in the department C intervention (“Coordination with other prevention campaigns”) and none in that of department A.

The key functions identified from the interventions delivered in the three departments differ strongly in relation to the following themes:

#### Interaction with teachers

All the interventions described possessing key functions aimed at facilitating the implementation of all or part of the intervention by teachers, either in the form of pre-structured sessions (department A) or in a free manner (departments B and C). In department B, the dieticians interact with teachers by providing support and information throughout the 2-year program (B3). In department A, the intervention is based on a “transfer of knowledge to teachers enabling them to conduct “turnkey” nutrition education sessions using a Teacher’s Guide” (A2). This transfer of knowledge takes the form of a 2-h training session for teachers on the key nutritional messages, and of the first nutrition education session being provided by the dieticians themselves in order to show the teachers how to deliver the sessions. In department C, on the other hand, the dieticians do not interact in any formal manner with the teachers and the information required for the implementation of the program by the latter is mainly provided in written form. The purpose is essentially to provide “materials for teachers containing nutrition information and guidance on implementing the program as extensively as they would like” (C5).

#### Adaptation to the school context

In department C, one of the key functions consists of the “use of teaching materials adapted to the “school format”” (C3). The teaching materials for the implementation of the “Eat and Move Well!” intervention have been designed by one teacher so that they integrate fully into the general objectives of the school curriculum. In this department, as in department A, the form of the materials and the activities offered to the pupils are similar to those normally used by teachers in the school context (A5). In department B, teacher adaptation takes the form of establishing a co-constructive relationship with the latter (B5): the dieticians do not provide materials or pre-designed sessions but help them to implement the program in the manner most appropriate to their context.

#### Promotion of physical activity

In department A, one of the key functions of the intervention targeted at pupils consists of a “delivery of nutrition education session using teaching materials that encourage knowledge building and experimentation in relation to physical activity” (A4), in the same format as the sessions focusing on food. In department C, the “physical activity” dimension of the intervention is more in-depth, and the key function consists of providing “teacher support for delivering physical education sessions allowing pupils to experiment with physical activity” (C4), using information sheets produced by the French Ministry for Education’s regional adviser on physical education and sport, and in association with the latter. The approach to physical activity in department B is completely different, with “physical activity encouraged through a collective, collaborative challenge” (B4).

#### Program continuity

The three interventions each contain a key function aimed at the continuity of the program within schools, but to very different degrees and in very different ways. In department B, continuity is inherent in the intervention because it aims to make sustainable changes to practices and is applied comprehensively within the school rather than in a limited manner (B7). In department A, the intervention explicitly includes “Remote support offered to schools that have already completed the action” (A9) without the interaction of dieticians with pupils (A9). Lastly, in department C, the key function described within this category is summarised as the “Option for schools to implement the program independently in subsequent years” (C9), by leaving them the teaching materials used to implement it.

Regarding the *context***,** it is important to point out that the three departments in which the “Eat and Move Well!” interventions have been described have different socio-economic and demographic characteristics. Departments B and C are particularly sparsely populated and contain many small schools, whereas department A, as well as being richer, is organised around a major city with larger educational establishments. The intervention history and institutional affiliation of the actors in question appear significant in the specificities of the key functions that we now observe in each department. As such, although it is now managed by a regional health promotion and education body (Instance régionale d’éducation et de promotion de la santé–IREPS), the “Eat and Move Well” intervention was, until 2014, delivered in department C by the Local health insurance bureau (*Caisse primaire d’assurance maladie*–CPAM), as in department A. This may partly explain why these two interventions seem to be more similar than department B’s intervention, managed by the IREPS from the start, and why they are more heavily based on standardised tools and contain key functions related to the screening for and management of overweight/obesity.

Context analysis also shows that changes to the national specifications and the funding terms for nutrition prevention interventions are translated differently according to the department, causing modifications of form and key functions. For example, intervention funding cuts have compelled actors in department A to rework their teaching materials to reduce printing costs, and actors in department C to reduce the attendance time of dieticians at local actor information meetings (see C7). Furthermore, the fact that the specifications mention the importance of physical activity in the approach to nutrition has led actors in department B to add a key function relating to encouraging pupils to exercise (B2).

Context, at a more local level, can also result in changes to the key functions of interventions, as we observed in department A. In 2015, the intervention was implemented in a school in a district where a cross-sectoral approach to the issue of health and a range of partnerships had already been established, enabling dieticians to introduce new key functions into their campaign (the provision of information to classroom assistants on nutrition messages, awareness-raising in canteen and out-of-hours childcare staff, and pupil-implemented changes to certain menus served in the canteen).

## Discussion

### Summary of principal results

The FIC description model was used to structure the description of the interventions in departments A, B and C and to define criteria for comparing the interventions. Our results show that, despite being produced from the same set of specifications and having similar objectives, the forms of these interventions varied. Likewise, although the key functions of the interventions in the three departments mostly reflected the same major themes, they did not overlap and were in some cases very different. Department B’s intervention was markedly different from those of the other two departments. The historical context of the interventions and the specificities of the local actors appear highly determinant of the key functions described, an observation consistent with other research demonstrating that local health policies are more influenced by local needs and the initial context than by national policy [21].

### Behind differences of form: different models of nutrition education and intervention

Comparison of the three departments’ interventions using the FIC model reveals implicit models of the intervention that are very different. These could be related to differing perceptions and considerations of the school as an implementation context for the nutrition education intervention. As such, actors in the three areas attempted to adapt to the teachers and to the school context, but did so in different ways. Schools have been described by several authors as “complex adaptive systems” [22]. These systems consist of individuals who can learn, interconnect, self-organise and co-evolve with their environment [23]. Some interventions have the capacity to take this complexity of context into account and to draw on its characteristics to produce their results [1]. They are not based on a fixed, pre-defined protocol but instead allow local actors great freedom to define the intervention and the form it takes in order to adapt to the context in which it is used [16]. The description of the key functions of department B’s intervention shows that it possesses some of the characteristics of this type of intervention, adapted to local complexities. The intervention conducted in this department is indeed based on a diagnosis of the school context (B2), it improves the ability of teachers to teach and self-organise through regular support (B3 and B4), it attempts to strengthen links and connections between different actors to create a network around the child (teachers, out-of-hours childcare staff, school canteen staff, etc.) (B10), it does not simply suggest “extra” activities but encourages the rethinking of routine activities (B6), and several of its key functions go beyond the subject of “nutrition”, acting in a more comprehensive fashion on factors that can impact various determinants of health (activities expanded to the entire school aimed at creating bonds between pupils (B7), increasing the participation of parents within since it allows the implementation context of the intervention to be taken into account, the field practitioners express that they lack the resources to describe it and to underline its value to those funding it, especially because it is difficult to distinguish this type of intervention from its implementation context and because it is not based on standardised tools. Traditional description and evaluation criteria may not be appropriate for this type of intervention. Interventions characterised by forms that are not defined in advance and that depend on interaction with the context to produce results have less predictable effects. However, they are potentially more important and more appropriate to the needs of local actors and the target population [24]. Various studies have shown that interventions based on the specific characteristics of “complex adaptive systems” were more effective than those that did not take the latter into account [23, 25] the school (B9)). This intervention model seems very attractive, and this approach could be effective even in small-scale interventions [26].

This result confirms that beyond individual behaviour changes, health interventions in schools can be conceived as interventions that enable teachers to build health promoting and supportive networks [27]. To this end, advocacy for longer interventions, with specifications allowing local actors to adapt them to their context, and more adaptive evaluation methods seems important.

### Benefits and limitations of the FIC approach

In this study, the choice was made to work co-constructively with those actually implementing the intervention on the ground, so that they could express themselves freely without the presence of their hierarchical superiors or those responsible for funding. However, this co-constructive process was only fully achieved for two of the three interventions studied. The independent dieticians from department C were only minimally involved in the process, mainly due to lack of time, but perhaps also because the intervention’s financial restrictions in this department resulted in the dieticians being distanced from decisions concerning the program [28]. Perhaps further consideration should be given in future studies to the financial compensation available for time spent on research. Furthermore, the dieticians in Department C operated in a relatively large rural geographical area, and the Transferiss research project could only offer them minimal compensation for travel.

The FIC approach also has various limitations, particularly regarding the degree of granularity of the key functions and the criteria for distinguishing between key functions and form, beyond the appraisal and negotiation of stakeholders. Some questions raised include the following: should there be a limit of only a few key functions per intervention? Conversely, would describing a program through a multitude of key functions be truly informative and enable its transferability?

Furthermore, although this approach allowed us to define criteria for comparing the interventions, the process raises an important issue: at what point one intervention can be considered similar to another or to constitute a totally different intervention. Although we can conclude from our results that department B’s intervention was very different from those of the other two departments, can we claim that the interventions of departments A and C constitute the same intervention even though there are many differences in their key functions?

The FIC approach has, however, enabled us to produce a detailed description of the interventions studied with the involvement of the implementing actors and to define criteria for comparing several interventions produced from the same set of specifications. It has proved suitable for complex real-life interventions routinely implemented, outside of any research context, allowing us to perceive differences that would not necessarily be apparent when looking only at institutional objectives or activities conducted as part of the campaign. Currently, some authors emphasise the major role played by local actors in the definition of what interventions’ element can be adapted or not [9, 29]. Indeed, they have legitimacy to change the intervention according to their own experiences and knowledge of their implementation contexts [29]. It is crucial for these actors to be reflexive and proactive to propose and monitor program’s adaptations [10]. In this context, providing structuring tools to support public health professionals in determining core elements of their intervention, like the FIC model, contribute to empower these actors [9].

### Distinguishing key functions and form to facilitate or understand the transfer of an intervention

Various tools have been designed in order to improve the description and reporting of public health interventions (e.g. TREND [30], CReDECI 2 [31], TIDieR [32] and ASTAIRE [33]). Recently, a simplified “meta-framework” has been created for structuring the description of interventions based on four categories: aims, ingredients, mechanisms and delivery [34]. Although these tools contain similar concepts, none of them, to our knowledge, refers to the distinction between key function and form. The definition of a key function overlaps partially but not completely with the definition of ingredients, defined as “the observable, replicable, and irreducible aspects of the intervention”, and that of mechanisms, defined as “the pathways or processes by which it is proposed that an intervention effects change or which change comes into effects” [34]. Furthermore, as the AIMD framework illustrates, few intervention description approaches recognise the importance of context [34]. The CICI (Context and Implementation of Complex Interventions) framework describes the links between the intervention, its setting, its context and its implementation [35]. The criteria used to describe these different categories are especially detailed for the context and implementation dimensions. The FIC model focuses on the description of the intervention and its context, but is less detailed on the implementation’s process in itself. It can also be used to complement other models and theories focusing on the description of the implementation process, its determinants or its evaluation [36]. However, the aim of the model is to provide a structure for joint reflection by various actors on the transferable aspects of the intervention, its form and its interactions with the context, in order ultimately to improve its potential transferability, rather than a checklist. Further research is needed to better define the conditions for the use of the FIC approach and to explore its acceptability and usefulness in other contexts.

## Conclusion

Our results confirm that the nutrition education interventions conducted in schools, although produced from a single set of national specifications, are adapted by local actors based on local context, history, and the relationships between these actors. For the interventions that we studied, some of the key functions varied greatly and translated different concepts of health education and modes of intervention to the population. It now seems vital to improve the description of interventions on the ground in order to highlight the key functions on which they are based, which still often remain implicit.

## Abbreviations

CICI: Context and Implementation of Complex Interventions
CNAMTS: National Employee Health Insurance Fund (In French: Caisse nationale d’assurance maladie des travailleurs salariés)
CPAM: Local health insurance bureau (In French: Caisse primaire d’assurance maladie)
FIC: Key functions, implementation, context
INCa: National cancer institute (In French: Institut National du Cancer)
IREPS: Health promotion and education body (In French: Instance régionale d’éducation et de promotion de la santé)
PNNS: National Health and Nutrition Program (In French: Programme national nutrition santé)
